# Vascular Factors and Multiple Measures of Early Brain Health: CARDIA Brain MRI Study

**DOI:** 10.1371/journal.pone.0122138

**Published:** 2015-03-26

**Authors:** Lenore J. Launer, Cora E. Lewis, Pamela J. Schreiner, Steve Sidney, Harsha Battapady, David R. Jacobs, Kelvin O. Lim, Mark D’Esposito, Qian Zhang, Jared Reis, Christos Davatzikos, R. Nick Bryan

**Affiliations:** 1 Laboratory of Epidemiology and Population Sciences, National Institute on Aging, Bethesda, MD, United States of America; 2 University of Alabama, Birmingham, AL, United States of America; 3 Division of Epidemiology and Community Health, University of Minnesota, Minneapolis, MN, United States of America; 4 Division of Research, Kaiser Permanente Northern California, Oakland, CA, United States of America; 5 Department of Radiology, University of Pennsylvania, Philadelphia, PA, United States of America; 6 Department of Radiology, University of Minnesota, Minneapolis, MN, United States of America; 7 Department of Radiology, University of California, Berkeley, Berkeley, CA, United States of America; 8 Division of Cardiovascular Sciences, National Heart, Lung, and Blood Institute, Bethesda, MD, United States of America; University Medical Center Rotterdam, NETHERLANDS

## Abstract

**Objective:**

To identify early changes in brain structure and function that are associated with cardiovascular risk factors (CVRF).

**Design:**

Cross-sectional brain Magnetic Resonance I (MRI) study.

**Setting:**

Community based cohort in three U.S. sites.

**Participants:**

A Caucasian and African-American sub-sample (n= 680; mean age 50.3 yrs) attending the 25 year follow-up exam of the Coronary Artery Risk Development in Young Adults Study.

**Primary and Secondary Outcomes:**

3T brain MR images processed for quantitative estimates of: total brain (TBV) and abnormal white matter (AWM) volume; white matter fractional anisotropy (WM-FA); and gray matter cerebral blood flow (GM-CBF). Total intracranial volume is TBV plus cerebral spinal fluid (TICV). A Global Cognitive Function (GCF) score was derived from tests of speed, memory and executive function.

**Results:**

Adjusting for TICV and demographic factors, current smoking was significantly associated with lower GM-CBF and TBV, and more AWM (all <0.05); SA with lower GM-CBF, WM-FA and TBV (p=0.01); increasing BMI with decreasing GM-CBF (p<0003); hypertension with lower GM-CBF, WM-FA, and TBV and higher AWM (all <0.05); and diabetes with lower TBV (p=0.007). The GCS was lower as TBV decreased, AWM increased, and WM-FA (all p<0.01).

**Conclusion:**

In middle age adults, CVRF are associated with brain health, reflected in MRI measures of structure and perfusion, and cognitive functioning. These findings suggest markers of mid-life cardiovascular and brain health should be considered as indication for early intervention and future risk of late-life cerebrovascular disease and dementia.

## Introduction

Identifying early risk factors and early changes in the brain will have a major impact on future clinical and public health priorities related to the looming epidemic of dementia. To reduce the incidence of dementia, different approaches will be needed to identify risky predictive changes in disease markers of the brain (outcome), as well as in putative risk factors (independent variable). Recent studies suggest pathologic processes start 10 to 20 years before clinical onset of dementia [1]. To better design prevention strategies, there is a call to study pre-clinical individuals to identify cerebral biomarkers that detect early disease. However, also needed, are studies of risk factor patterns and the concurrent cerebral characteristics that initiate pathologic processes and may indicate pre-clinical risk for dementia [2]. This question can be addressed in well described longitudinally followed community based cohorts with a wide range of health characteristics Yet, as recently reviewed literature on the prevention of Alzheimer’s disease [3], and on the associations of CVRF to a range of MRI sequences and modalities [4], such data are lacking on middle age community dwelling individuals, and in minority populations. As reviewed, studies relating CVRF to multiple MRI characteristics, are based on small samples, patient populations with particular diseases, or community based cohorts who are older than 60 years. Further, investigations into the association of CVRF to MRI markers have focused on volumetric outcomes, such as total brain volume (TBV) and burden of white matter disease measured by lesion load, which are thought to be later manifestations of disease.

With the aim of establishing CVRF-MRI correlations at an earlier age then has previously been studied, we examine the association of MRI brain outcomes to selected CVRF in a well described middle age bi-racial cohort, whose brain has been characterized by multiple Magnetic Resonance Imaging (MRI) sequences that reflect brain structure, physiology and function. Although many candidate CVRF have been identified as associated with MRI brain outcomes [5], here we focus on smoking, sedentary activity, hypertension, diabetes and obesity. These factors have been *a priori* selected because their associated risks for dementia have been replicated in several cohort studies with a wide range of demographic groups [5,6], and because they are important components of the public’s current CVD risk profile [7]. We examine the association of these selected CVRF to TBV, and white matter disease, as well as measures of cerebral perfusion and brain tissue microstructural integrity These latter two characteristics have been suggested to provide indicators of early changes in the brain that proceed to, or form the anatomical basis of, dementia [8,9], [10], [11, 12].

## Methods

### Study population

Data are from a sub-sample of black and white men and women who participate in the community- based Coronary Artery Risk Development in Young Adults (CARDIA) Study (baseline 1985) and were examined (2010) at a mean age of 50 years. The CARDIA Study [13] is a longitudinal study of the development and determinants of cardiovascular disease in 5,115 young adults aged 18–30 years at baseline in 1985–1986. The community based sample was recruited from four US cities (Birmingham, Alabama; Chicago, Illinois; Minneapolis, Minnesota; and Oakland, California) to be approximately balanced within center by sex, age (18–24 years and 25–30 years), race (white, black), and education (≤high school, >high school) [13]. In 2010 ― 2011, 72% of the surviving cohort attended the 25 year (Y25) follow-up exam. All participants provided written informed consent at each exam, and institutional review boards from each field center and the coordinating center (The University of Alabama Birmingham Institutional Review Board, University of Minnesota Institutional Review Board, Kaiser Permanente Northern California Institutional Review Board), annually approve this study.

As a part of the Y25 exam, a sub-sample of the cohort participated in the CARDIA Brain MRI sub-study. This sub-study was designed to characterize the morphology, pathology, physiology and function of the brain with magnetic resonance imaging (MRI) technology. The sample for the CARDIA Brain MRI sub-study was enrolled at the time Y25 appointments were made, with the aim of achieving a balance within four strata of ethnicity/race (black, white) and sex from three of the CARDIA field centers: Birmingham, AL, Minneapolis, MN, and Oakland, CA. Each center had a Brain MRI s target sample size and when reached enrollment was ended. Exclusion criteria at the time of sample selection, or at the MRI site, were a contra-indication to MRI or a body size that was too large for the MRI tube bore.

Separate written consent for participation in the Brain MRI sub-study was obtained, and separate approval was given by the IRBs governing participating sites (The University of Alabama Birmingham Institutional Review Board, University of Minnesota Institutional Review Board, Kaiser Permanente Northern California Institutional Review Board, University of Pennsylvania Institutional Review Board, and the NIH Office of Human Subjects Research Protection for the Intramural Research Program, National Institute on Aging).

### MRI acquisition and processing

Brain MRI were acquired on 3-T MR scanners located proximal to each CARDIA clinic site (UCB: Siemens 3T Tim Trio/VB 15 platform; UMN: Siemens 3T Tim Trio/VB 15 platform and UAB: Philips 3T Achieva/2.6.3.6 platform). The MRI Reading Center (RC), located at the University of Pennsylvania (Dr. RN Bryan), worked in collaboration with the MRI field centers to train technologists to standardized protocols, and transfer MRI data to a central archive located at the MRI RC. To evaluate scanner stability and image distortion prior to site acceptance and quarterly thereafter, each MRI field center followed standard quality assurance protocols developed for the Functional Bioinformatics Research Network (FBIRN), and the Alzheimer's disease Neuroimaging Initiative (ADNI). The following established quality assurance acceptance thresholds were used: FBIRN—Siemens scanners SFNR >220, RDC>3.1, Philips scanners SFNR>220, RDC>2.4; ADNI—SNR >300, Maximum Distortion >2.0. Performance across the scanners was acceptable for all sequences except the pCASL acquired at UAB so perfusion data from this site is not reported.

Parameters of interest were estimated as follows: From the sagittal 3D T1 sequence (Plane Sagittal Coil 12channel File name 3D T1 MPRAGE: Tr 1900 Te 2.89 Fov 250mm, thickness 1mm slices 176 slices, Base Res 255, Phase res 100%, Matrix 256X256 NSA 1 TI 900 ms Pixel BW 170hz. ETL = 1 Flip = 9), we estimated total intracranial volume (TICV), (a measure of head size) as the sum of gray matter (GM),white matter (WM) and cerebral spinal fluid (CSF) volumes, and total brain tissue volume (TBV) as the sum of GM and WM volumes). We estimated abnormal white matter tissue from the sagittal 3D FLAIR (Plane Sagittal Coil 12channel File name 3D FLAIR: Tr 6000 Te 160 Fov 250mm (fov phase = 85%), thickness 1 mm slices 160 slices, Base Res 202, Phase res 91%, Matrix 258 X 221 NSA 1 TI = 2200 ms Pixel BW 930, ETL 203)), T1 and T2 (Plane Sagittal Coil 12 channel Psd File name 3D T2: Tr 3200 Te 409 Fov 250 (fov phase = 80%), thickness 1mm slices 176, Base Res = 246, Phase res = 80%, Matrix 258x256 NSA 1 Center Freq. water ETL 141 Flip 120 Pixel BW 750)[14] sequences. Brain microstructural tissue integrity and organization were estimated from axial Diffusion Tensor Images (DTI); Plane Axial Coil 12-channel Psd File name ep2d diff MDDW: TR 7300 TE 84 Fov 245 Thickness 2.2mm distance factor 0% Diff Directions- 33 Concatenations = 1 number sl = 64 Flip 90 Matrix 128x128 NSA 1 Center Freq. water Phase FOV = 100 Pixel BW = 1860 diff mode = Free Phase part fourier = 7/8 Echo spacing = .59 diff weighting = 1 Accel factor = 3 EPI factor = 112 Base Res 112 Phase res 100%; 2 times). Here we report on the WM-DTI—derived fractional anisotropy (FA) measure, which ranges from 0 to 1 and estimates the degree (or uniformity) to which water diffuses along the direction of myelinated tracks in the white matter [10, 14, 15]; Zero indicates equal probability of diffusion in all directions (i.e. there is no structural restriction to the flow of molecules), and a ‘1’ indicates the diffusion occurs along one axis (i.e., the WM tract). Cerebral brain perfusion (CBF; volume of flow per unit brain mass per unit time (mL/100g/min)) was measured with an axial pseudo-Continuous Arterial Spin Labeling (pCASL) technique (Plane Axial Coil 12 channel Psd File name pCASL: Tr 4000 Te 11 Fov 220 mm, Concatenations 1 number sl 20, Base Res 64, Phase res = 100%, thickness 5, distance factor 20%, Center Freq. water, Matrix 64 x 64 NSA 1 Fat suppression = ON Flip = 90 Echo spacing = .47) [11, 12]. Here we present the estimate for the GM as it is more reliably obtained than measures in WM. Per participant, completion rates for the sequences ranged from 95.9% (pCASL) to 100% (T1, PD/T2 and FLAIR sequences).

A graded alert system was established for patient safety. If the MR technician detected pathology that needed immediate attention, the site PI and radiologist were immediately notified and appropriate action taken. Otherwise, each site followed standard operating procedures that involved a clinical reading of the scan by a local clinical and the MR RC within 48 hours.

Image processing was performed by the Section of Biomedical Image Analysis, Department of Radiology, University of Pennsylvania (Dr. C. Davatzikos). Before starting the processing pipeline, an initial QC protocol identified any motion artifacts or any other quality issues; images that failed this QC test were flagged for inspection. After this QC procedure, the scans were processed through an automated pipeline. Quality checks were performed on intermediate and final processing steps by visual inspection and by identifying outliers of calculated variable or parameter distributions.

Structural MR images, processed using previously described methods [16–18] [19], were based on an automated multispectral computer algorithm that classifies all supratentorial brain tissue into GM, WM, and CSF. GM and WM were further characterized as normal and abnormal and then into specific regions of interest (98 in the normal tissue and 94 in the abnormal tissue). Abnormal WM includes tissue damage due to ischemia, demyelination and inflammation, as well as the damaged penumbra tissue surrounding focal infarcts. Since the amount of abnormal gray matter, which includes infarcted cortical tissue, was very low (average: 0.17―0.35 cc., median: 0.1 cc.), we do not report these data separately. Standard methods for calculating FA from the raw DTI images were used to derive voxel-wise maps [20]. Average FA was computed using the anatomical regions of interest in WM-FA. The mean perfusion volume from the pCASL was quantified into CBF units using the model and software described in Wang et al. [21], resulting in a CBF map. The technical error of measurement, an accuracy index that reflects measurement quality of both acquisition and processing of scans, was estimated from scans of 3 persons measured 3 times in the 3 centers; results were 1.2% for TBV, 27.8% for Abnormal WM, 3.4% for FA-WM and 7.3% for GM-CBF.

### Risk factors

The behavioral and clinical risk factor data reported here were collected in the Y25 exam, concurrently with the MRI. Seated blood pressure (BP) levels were measured with an OmROn Hem907XL sphygmomanometer on the right arm three times by a trained technician; measures two and three were averaged and used for the blood pressure analysis. Readings were categorized into normotensive (SBP <130/DBP <85 mmHg and no treatment), pre-hypertensive (130–139/85 - <90 mmHg), and hypertensive (≥140/≥90 mmHg or treatment)[22]. Diabetes was defined following ADA criteria [23] for levels of fasting, non-fasting or postprandial OGTT results, HbA1c percent, or use of anti-diabetes medication; smoking (never, former, current) was assessed by questionnaire. Participants were also questioned about weekly hours engaged in sedentary activities, including listening to TV or music, doing deskwork, talking on the phone, or driving [24]. To capture more extreme comparisons we categorized weekly hours into <25 percentile (i.e. *least* amount of sedentary time; (<4.3 hrs), 25–75 percentile, and ≥ 75 percentile (> 8.5 hrs). Body Mass Index (BMI) was calculated from measured height (to the nearest 0.5 cm) and weight (to the nearest 0.2 kg), and categorized according to WHO [25] criteria into normal (<25 km/m^2^), overweight (25 - <30), obese (30 - <35) and very obese (≥ 35).

### Cognition

To determine whether the variation in MRI characteristics had functional consequences we investigated the association between MRI values and a composite cognitive score composed of three cognitive tests, described previously [26], administered in the exam concurrent with the MRI: Digit Symbol Substitution Test (measure of psychomotor speed) scored as the number of digits correctly substituted [27], Rey Auditory Verbal Learning Test (verbal memory), scored as the total number of words recalled in the immediate and delayed recall [28], and the modified Stroop test (executive function), scored as the total seconds plus errors to complete the interference test where the participant reads a color word that is printed in another color (higher score is worse performance) [29]. The tests were administered by trained and certified CARDIA technicians following the protocol of previous studies [30]. The composite score was calculated by transforming raw test scores into Z-scores ((individual score—group mean score)/SD) and adding them together.

### Statistical Analyses

There were 719 participants who participated in the BRAIN study: 35% (n = 252) from Oakland, 41.3% (n = 297) from Minnesota, and 23.6% (n = 170) from Birmingham. Of these, 680 had successfully processed images and complete risk factor data, including 517 with brain perfusion data. All brain-related variables were transformed into Z scores (as above), so results could be compared across sequences on a standardized one-SD increment.

Since many trials are designed to intervene on one risk factor, we show the results of a single CVRF (smoking, sedentary activity, (pre) hypertension, diabetes, and obesity), adjusted for demographic variables (age, sex, race and education). In a supplementary table we report the full model that includes all the variables together for each of the four MRI sequences. All models for TBV, AWM, and WM-FA were adjusted for ICV, as a measure of head size; models for CBF were adjusted for TBV, as a measure of total tissue perfused.

## Results

The mean age of the sample was 50.3 (SD 3.5) years, 52.2% were female and 39.4% were Black. Mean systolic and diastolic blood pressures were within the normal range, but overall, the sample had a relatively ‘risky’ cardiovascular risk factor profile: 35.9% had a BMI of 30 or higher, 32.2% had hypertension, and 39.4% had a history of smoking (Table 1). 37% had 3 or more risk factors, most commonly hypertension, BMI over 30 and smoking After adjusting for age and sex, correlation among the sequences themselves ranged from 0.001 to 0.36 (S1 Table).

**Table 1:** Demographic, behavioral, clinical and brain characteristics in a bi-racial middle-age cohort:CARDIA BRAIN Sub-study.

	N	%	Mean (SD)
**Age (y)**			**50.3 (3.5)**
**Sex**			
**Male**	**325**	**47.8**	
**Female**	**355**	**52.2**	
**Race**			
**White**	**412**	**60.6**	
**Black**	**268**	**39.4**	
**Education (y)**			
**<12**	**22**	**3.2**	
**12**	**119**	**17.5**	
**13–16**	**400**	**58.8**	
**>16**	**139**	**20.4**	
**Smoking**			
**Never**	**412**	**60.6**	
**Former**	**161**	**23.7**	
**Current**	**107**	**15.7**	
**Sedentary time (hrs)**			**6.8 (4.1)**
**< = 25th p (< 4.3 hrs)**	**172**	**25.3**	
**>25 - < = 75 p (4.3 - <8.5 hrs)**	**345**	**50.7**	
**>75th p (>8.5 hrs)**	**163**	**24.0**	
**Body Mass Index**			**28.7 (5.7)**
**<25**	**201**	**29.6**	
**25-<30**	**235**	**34.6**	
**30-<35**	**140**	**20.6**	
**> = 35**	**104**	**15.3**	
**Blood pressure**			
**SBP, mmHG**	**680**		**118.1 (14.7)**
**DBP, mmHG**	**680**		**73.5 (10.7)**
**Normotensive**	**384**	**56.5**	
**Pre-hypertension**	**77**	**11.3**	
**Hypertension**	**219**	**32.2**	
**Diabetes**			
**No**	**611**	**89.9**	
**Yes**	**69**	**10.2**	
**Number behavioral and clinical risk factors** ^**a**^			
**0**	**79**	**11.6**	
**1**	**194**	**28.5**	
**2**	**152**	**22.4**	
**3+**	**255**	**37.5**	
**Brain characteristics**			
**Total Brain Volume (cm** ^**3**^ **)**	**680**		**985.5 (105.8)**
**AWM (cm** ^**3**^ **)**	**680**		**0. 3 (0.1, 0.6)** ^**c**^
**GM-CBF (mL/100g/min)**	**517**		**56.0 (11.3)**
**WM-FA**	**680**		**0.3 (0.02)**
**Composite cognitive score** ^**b**^	**663**		**0.1 (2.3)**

Abbreviations: SBP, systolic blood pressure; DBP, diastolic blood pressure; DSST, Digit Symbol Substitution Test; AWM, abnormal white matter; GM-CBF, gray matter cerebral blood flow; WM-FA, white matter fractional anisotropy.

^a^ Includes Smoking, Sedentary activity (upper 75th p); BMI >30; Diabetes, and Hypertension.

^b^ Composite is the sum of Z-scores from the DSST, modified Stroop Test and Rey Auditory Verbal Learning Test.

^c^ median (25%, 75%).

### Demographic factors

Even within the relatively narrow age (range 43 to 55 y) of the sample, there was already a detectable negative association between age and TBV, WM-FA, and GM-CBF (Table 2). Adjusting for sex, race and ICV, TBV was 1.058 cc (0.01 SD) lower for each year of age. Adjusting for ICV, age and race, males had significantly lower GM-CBF per mL/g/min. Compared to Whites, Blacks had more AWM, and higher GM-CBF. Education level was not associated with detectable differences in any of the MRI brain characteristics.

**Table 2:** Brain characteristics by demographic, behavioral and clinical measures in a bi-racial middle-age cohort: CARDIA Brain Sub-study.

	TBV		AWM		WM-FA		GM-CBF	
	Effect^a^	p	Effect^a^	p	Effect^a^	p	Effect^a^	P
**Age**								
Slope (y)	-0.01 (0.003)	<.001	0.002 (0.011)	0.88	-0.03 (0.011)	0.008	-0.03 (0.012)	0.005
<50 y	0.05 (0.02)	<.001	0.02 (0.06)	0.97	0.11 (0.06)	0.02	0.19 (0.07)	0.003
50+y	-0.03 (0.01)		0.01 (0.05)		-0.07 (0.05)		-0.07 (0.05)	
**Sex**								
Male	-0.02 (0.02)	0.06	-0.07 (0.06)	0.09	-0.07 (0.06)	0.15	-0.32 (0.06)	<.001
Female	0.02 (0.02)		0.10 (0.06)		0.06 (0.06)		0.37 (0.06)	
**Race**								
Black	0.003 (0.017)	0.78	0.096 (0.064)	0.06	-0.013 (0.061)	0.84	0.206 (0.069)	<.001
White	-0.004 (0.014)		-0.068 (0.051)		0.004 (0.049)		-0.152 (0.052)	
**Education (y)** ^**b**^								
< 12	0.05 (0.06)	0.32	-0.10 (0.21)	0.67	-0.19 (0.20)	0.33	0.04 (0.24)	0.65
12	0.01 (0.02)	0.41	-0.08 (0.09)	0.53	-0.01 (0.09)	0.72	0.03 (0.10)	0.36
13–16	-0.003 (0.01)	0.66	0.057 (0.05)	0.58	-0.001 (0.05)	0.74	-0.019 (0.05)	0.08
>16	-0.02 (0.02)	ref	0.0007 (0.09)	ref	0.03 (0.08)	ref	0.16 (0.09)	ref
**Smoking** ^**b**^								
Never	0.01 (0.01)	ref	-0.07 (0.05)	ref	0.06 (0.05)	ref	0.08 (0.05)	ref
Former	0.01 (0.02)	0.83	0.07 (0.08)	0.14	-0.04 (0.08)	0.24	-0.16 (0.08)	0.01
Current	-0.05 (0.03)	0.04	0.25 (0.10)	0.004	-0.18 (0.09)	0.02	0.11 (0.10)	0.79
**Sedentary time** ^**b**^								
Slope (hrs)	-0.004 (0.003)	0.09	-0.009 (0.010)	0.37	-0.021 (0.009)	0.02	-0.017 (0.011)	0.13
>75th percentile	-0.06 (0.02)	0.01	-0.04 (0.08)	0.13	-0.14 (0.07)	0.05	-0.09 (0.09)	0.08
25th- 75th	0.02 (0.01)	0.98	-0.02 (0.06)	0.10	0.03 (0.05)	0.55	0.04 (0.06)	0.37
**Body mass index** ^**b**^								
Slope (kg/m2)	0.001 (0.002)	0.42	-0.002 (0.007)	0.77	-0.006 (0.007)	0.36	-0.023 (0.007)	0.002
<25	-0.009 (0.020)	0.93	-0.012 (0.074)	0.42	0.068 (0.070)	0.28	0.226 (0.075)	0.09
25 - <30	-0.007 (0.017)	ref	0.067 (0.066)	Ref	-0.032 (0.062)	ref	0.058 (0.067)	ref
30 - <35	0.02 (0.02)	0.42	-0.01 (0.08)	0.49	-0.01 (0.08)	0.80	-0.12 (0.09)	0.10
35+	0.006 (0.03)	0.68	-0.031 (0.10)	0.41	-0.069 (0.09)	0.74	-0.226 (0.11)	0.03
**Blood Pressure** ^**b**^								
Systolic BP (mmHg)	-0.0008 (0.001)	0.28	0.0084 (0.003)	0.002	-0.0076 (0.003)	0.004	-0.0045 (0.003)	0.13
Diastol BP mmHg)	-0.0010 (0.001)	0.31	0.0066 (0.004)	0.08	-0.0080 (0.004)	0.03	-0.0098 (0.004)	0.01
Normal	0.02 (0.01)	ref	-0.06 (0.05)	Ref	0.06 (0.05)	ref	0.12 (0.06)	ref
Pre-hypertension	0.01 (0.03)	0.67	-0.06 (0.11)	0.98	-0.01 (0.11)	0.52	-0.20 (0.12)	0.01
Hypertension	-0.04 (0.02)	0.009	0.15 (0.07)	0.02	-0.11 (0.06)	0.04	-0.06 (0.07)	0.05
**Diabetes** ^**b**^								
No	0.01 (0.01)	0.007	-0.01 (0.04)	0.14	-0.002 (0.04)	0.86	0.03 (0.04)	0.82
Yes	-0.08 (0.03)		0.18 (0.12)		-0.02 (0.11)		0.00 (0.14)	
**Composite cognitive score** ^**c**^	0.016 (0.006)	0.005	-0.056 (0.021)	0.009	0.061 (0.020)	0.003	0.041 (0.017)	0.02

Abbreviations: TBV, total brain volume; AWM, abnormal white matter; GM-CBF, gray matter cerebral blood flow; WM-FA, white matter fractional anisotropy; BP, blood pressure; DSST, Digit Symbol Substitution Test.

^a^ Values are adjusted Mean Z-scores of brain characteristics or the slope (β) of the change in brain Z-score by unit of the independent variable.

^b^ All models are adjusted for age, sex, and race.

^c^ Also adjusted for education.

### Behavioral factors

In age, sex and race adjusted models, smoking behavior was strongly associated with indicators of brain pathology: Current smokers, compared to never smokers, had significantly smaller TBV, more AWM and lower WM-FA indicating less tissue integrity. Former smokers had similar brain characteristics to never smokers, except for GM-CBF, which was lower in former compared to never smokers. There was a monotonic decrease in all of the MRI outcomes, with TBV and WM-FA being significantly lower in persons in the 75^th^ compared to lowest 25th percentile of time spent in sedentary activities (Table 2).

### Clinical factors

Hypertension was clearly associated with indicators of diminished brain health. Expressed as continuous measures, increasing systolic blood pressure was significantly associated with increasing AWM and decreasing WM-FA; increasing diastolic blood pressure was associated with significantly lower WM-FA and GM-CBF. These middle age participants with hypertension had significantly smaller TBV, more AWM tissue, lower WM-FA and lower GM-CBF compared to normotensive persons. Persons with pre-hypertension levels of blood pressure had significantly lower GM-CBF. Compared to those with no diabetes, persons with diabetes had overall less healthy brains, but only their TBV was significantly smaller (Table 2). Results of the multivariate analyses that included all the risk factor information were essentially the same as the age, sex and race adjusted models (S2 Table).

### Cognitive function

Variation in brain characteristics was significantly associated with cognitive function score. Controlling for age, sex, race, education, and ICV, the composite cognitive score was significantly (Table 2) associated with less pathology: higher TBV, less AWM, higher WM_FA and moderately higher GM-CBF. Results of individual tests are shown in S3 Table.

## Discussion

In this bi-racial middle age cohort, we found variations in brain characteristics that were associated with age and key modifiable clinical and behavioral indicators of a healthy CV profile. The trends in brain characteristic—risk factor associations may be suggestive of incipient or already established cerebral pathology, including smaller TBV, more AWM, lower WM-FA and lower GM-CBF. In general, the mean values of the MRI measures are, of course, higher than in older persons with clinical dementia, and possibly those with pre-clinical dementia. Never-the-less there were significant associations of CVRF to MRI measures, and MRI measures to cognition [26], suggesting the MRI measures we investigated may be relevant markers for later risk of cognitive decline. Since the sub-sample of participants in the Brain MRI sub-study mirrored the total CARDIA cohort, and is similar to the US NHANES III nationally representative sample in smoking history, diabetes, and mean blood pressures [31], it is reasonable to suggest the findings we report here are relevant to evaluating brain health in White and Black nationally representative samples in NHANES.

This study has several strengths. It provides the first data on a large community-based bi-racial cohort of this age (mean 50 years), of a range of MRI characteristics in relation to key risk factors for cerebrovascular and coronary disease, as well as for late-life dementia. In particular there are very few studies that have described cross-sectional associations of these CVRF to FA and GM-CBF. Such studies on this age cohort are important as they will provide clues of early changes in the brain, that may eventually predict who is at risk for dementia. Indeed, its cross-sectional study design is a limitation, and an additional follow-up is planned.

Values of TBV among 80 year olds with dementia range between 70%-75% [9] of total intracranial volume, compared to the CARDIA mean of 81% in 50 year olds. Studies of older cohorts have found no difference [32] or a higher TBV in Black compared to White participants; for example in the multi-ethnic WHICAP cohort, the difference was 1.6%, compared to a 0.41% in CARDIA [33]. In WHICAP, compared to Whites, Blacks had a significantly higher load of white matter vascular changes, whereas in CARDIA AWM was higher, but not significantly, in Blacks than Whites. Data on perfusion differences in community-dwelling Black and White subjects are scant. We found higher perfusion rates in Blacks compared to Whites. Possibly, the higher perfusion rates in Blacks compared to Whites reflects compensatory flow in the context of vascular disease, but this awaits follow-up data. Finally, the finding of no differences by education in these multiple brain characteristics is of interest because lower educational attainment has often been associated with poorer scores on cognitive tests and a higher risk for dementia [34]. The different conclusions regarding education from these imaging findings compared to studies of cognitive function suggest one or several variable(s) explaining the gap are ‘missing ‘ and require further investigation.

We found current smoking is associated with multiple measures of the brain, ranging from decreased WM-FA, increased AWM, and smaller TBV. We also found former smokers had the lowest GM-CBF that was significantly different from never smokers. Former smokers are usually a mixed group of those who stopped smoking to prevent future disease, or stopped smoking because of existing disease. More detailed investigations into smoking history and its effects on the brain are warranted.

We show, for the first time, that sedentary behavior [35], may pose its own risks to brain health. There is increasing evidence that sedentary behavior, separate from shorter bouts of moderate physical activity, increases the risk for cardio-metabolic disturbances and disease. Based on the emerging evidence, suggestions for CVD risk reduction have been made to intervene to *decrease sitting*, rather than to increase moderate exercise, which may be difficult for at-risk persons to adopt [19]. Such strategies may be beneficial for brain health, and should be further explored.

We found a significant association of very high BMI, indicating very obese, to lower GM-CBF and no other MRI outcomes. BMI related metabolic or vascular factors, generated peripherally or centrally, have been postulated to increase the risk for dementia [36]. Our data suggest, if there is an association, the cerebral tissue changes may appear later secondary to reduced cerebral perfusion. It is also possible, our finding reflects the breathing and sleeping problems that accompany very high BMI [37], which is a different pathway to cognitive problems than previously suggested for high BMI.

Mid-life high blood pressure has been associated with stroke, dementia, white matter lesions, and neuropathologic lesions consistent with neurodegeneration and vascular disease [36]. We show that in this representative middle age community-cohort, age 50 years, persons with hypertension have lower TBV, WM-FA, GM-CBF and more AWM, all suggesting poorer brain health compared to normotensives. These findings strongly suggest control of blood pressure in mid-life may be important to reducing unwanted early brain changes. The point at which high blood pressure should be treated however, is still under consideration. The newest guidelines recommend initiating treatment in persons 60 years and older with a systolic blood pressure ≥ 150 mmHg [38]. However, we found that, as a continuous variable, increasing blood pressure is associated with relatively poorer brain health in mid-life, reinforcing the need to evaluate guidelines for treatment specifically for cerebral outcomes.

The relationship of diabetes to metabolic and vascular changes leading to late age dementia, while replicated in several studies [39], remains controversial. We found a significantly smaller TBV in persons with, compared to those without diabetes. This is consistent with a finding of reduced gray matter with an increasing duration of diabetes in persons with long standing diabetes, average 62 years [40]; as well as in older men and women participating in the AGES-Reykjavik Study [30]. Also similar to both studies, there was no association with WML, which was expected due to vascular changes in T2D. Possibly, diabetes leads directly to atrophic patterns, which are followed at a later time, by secondary WM diffuse damage.

As differences per year of age is often used as a metric to evaluating clinical significance of differences by risk factors, it is of interest to note that in this middle age cohort, differences in TBV by some risk factors are similar or greater than the 0.08 difference in TBV between the <50 yr old strata and ≥50 yr old strata. For example, there is a 0.04 SD difference between persons with normal blood pressure and hypertension, a 0.05 SD difference between never and current smokers, a 0.10 SD difference between persons with compared to without diabetes and 0.09 SD difference between those spending a relatively high vs. low amount of time in sedentary activities.

Our findings on risk factors are generally consistent with mid-to-late life studies investigating risk factors for cognitive disorders. However the mid to late life studies are based on cohorts of participants who were identified and examined (but not imaged) in mid-life and survived to be evaluated in a late-life exam. That study design allows investigation into survivors but does not allow the study of relationships going forward from baseline, as CARDIA does, so that valid early markers of brain disease can be identified. In addition these previous studies have not acquired DTI or perfusion data.

In middle age, compared to older age where comorbidity is an issue, contrasts by CVRF may be sharper and we can better track how these factors may become risk factors for future dementia. Longitudinal follow-up of these individuals, and others who participate in long term studies, is needed to further characterize intermediary trajectories in both CVRF and MRI outcomes.

We hypothesize that together, these measures reflect a ‘pathologic’ cyclic cascade whereby the perfusion decreases, followed by decreased tissue integrity, and then macroscopic changes due to neurodegeneration or vascular damage. Although this is a cross-sectional study we cannot draw conclusions on the temporal relationships among levels or change in the MRI measures, there are several important clinical and public health messages that can be taken from these findings now: 1) There is suggestive evidence that cognitive differences and brain pathology are associated in this current cohort of middle-age individuals; 2) Modifiable mid-life risk factors are associated with mid-life brain health; 3) Changes in life style factors, such as stopping smoking and reducing time spent in sedentary activity, may directly or indirectly via hypertension and diabetes, improve brain health; 4) Relatively simple and already known methods to reduce hypertension, in particular, should be targeted. In conjunction with evidence from late-life studies, we propose early intervention may reduce late-life cognitive disorders that impair an individual’s quality of life and drive up health care costs.

## Supporting Information

S1 Table Partial Correlation Coefficients among MRI sequences. (DOCX)

S2 Table Full model of correlates of brain characteristics in a bi-racial middle-age cohort: CARDIA BRAIN sub-study. (DOCX)

S3 Table Associations of individual tests of cognition and multiple measures of brain health: CARDIA BRAIN sub-study. (DOCX)
