# Supplementary material for: Vascular Factors and Multiple Measures of Early Brain Health: CARDIA Brain MRI Study
Source: PLoS One. 2015 Mar 26;10(3):e0122138. doi: 10.1371/journal.pone.0122138 (PMC4374951; doi:10.1371/journal.pone.0122138)
Supplement: S1 Table — (DOCX) [file pone.0122138.s001.docx]

| S1 Table. Partial Correlation Coefficients among MRI sequences. | | | | | |
| --- | --- | --- | --- | --- | --- |
| CARDIA BRAIN Sub-study | | | | | |
|  | TBV | AWM | WM-FA | GM-CBF |  |
| TBV | 1 | 0.02 | 0.36* | -0.07 |  |
| AWM |  | 1 | 0.14* | - |  |
| WM-FA |  |  | 1 | - |  |
| * p<0.0002, adjusted for age and sex | | | | | |
