# Supplementary material for: Vascular Factors and Multiple Measures of Early Brain Health: CARDIA Brain MRI Study
Source: PLoS One. 2015 Mar 26;10(3):e0122138. doi: 10.1371/journal.pone.0122138 (PMC4374951; doi:10.1371/journal.pone.0122138)
Supplement: S2 Table — (DOCX) [file pone.0122138.s002.docx]

S2 Table. Full model of correlates of brain characteristics in a bi-racial middle-age cohort: CARDIA BRAIN Sub-study.

|  | **TBV** |  | **AWM** |  | **WM-FA** |  | **GM-CBF** |  |
| --- | --- | --- | --- | --- | --- | --- | --- | --- |
|  | Effect | P | Effect | P | Effect | P | Effect | P |
| **Age** |  |  |  |  |  |  |  |  |
| Slope (y) | -0.01 (0.003) | <.001 | -0.01 (0.011) | 0.52 | -0.03 (0.011) | 0.01 | -0.03 (0.012) | 0.04 |
| **Sex** |  |  |  |  |  |  |  |  |
| Male | -0.05 (0.03) | 0.21 | -0.03 (0.10) | 0.02 | -0.12 (0.10) | 0.36 | -0.35(0.12) | <.001 |
| Female | -0.015 (0.03) |  | 0.20 (0.10) |  | -0.03 (0.10) |  | 0.39 (0.12) |  |
| **Race** |  |  |  |  |  |  |  |  |
| Black | -0.019 (0.027) | 0.30 | 0.178 (0.101) | 0.05 | -0.040 (0.096) | 0.48 | 0.291 (0.114) | <.001 |
| White | -0.045 (0.027) |  | -0.003 (0.101) |  | -0.103 (0.096) |  | -0.250 (0.117) |  |
| **Education (y)** |  |  |  |  |  |  |  |  |
| < 12 | 0.01 (0.06) | 0.17 | -0.04 (0.22) | 0.37 | -0.15 (0.21) | 0.71 | 0.06 (0.26) | 0.92 |
| 12 | -0.02 (0.03) | 0.15 | 0.002 (0.11) | 0.19 | -0.02 (0.11) | 0.73 | 0.02 (0.13) | 0.66 |
| 13-16 | -0.048 (0.02) | 0.37 | 0.207 (0.08) | 0.75 | -0.055 (0.08) | 0.93 | -0.076 (0.10) | 0.12 |
| >16 | -0.07 (0.03) | ref | 0.18 (0.11) | ref | -0.06 (0.11) | ref | 0.08 (0.12) | ref |
| **Smoking** |  |  |  |  |  |  |  |  |
| Never | -0.014 (0.03) | ref | -0.09 (0.10) | ref | 0.03 (0.09) | ref | 0.09 (0.11) | ref |
| Former | -0.009 (0.03) | 0.83 | 0.08 (0.11) | 0.07 | -0.07 (0.11) | 0.29 | -0.17 (0.13) | 0.009 |
| Current | -0.07 (0.03) | 0.05 | 0.28 (0.12) | 0.001 | -0.18 (0.12) | 0.05 | 0.14 (0.13) | 0.67 |

S2 Table. (Continued).

|  | TBV |  | AWM |  | WM-FA |  | GM-CBF |  |
| --- | --- | --- | --- | --- | --- | --- | --- | --- |
|  | Effect | P | Effect | P | Effect | P | Effect | P |
| **Sedentary time** |  |  |  |  |  |  |  |  |
| >75th percentile | -0.08 (0.03) | 0.02 | -0.02 (0.11) | 0.03 | -0.17 (0.11) | 0.16 | -0.04 (0.13) | 0.42 |
| 25th - 75th | -0.01 (0.03) | 0.88 | 0.04 (0.09) | 0.03 | -0.03 (0.09) | 0.87 | 0.03 (0.11) | 0.76 |
| <25 percentile | -0.01 (0.03) | ref | 0.24 (0.12) | ref | -0.01 (0.11) | ref | 0.06 (0.13) | ref |
| **Body mass index** |  |  |  |  |  |  |  |  |
| <25 | -0.059 (0.030) | 0.71 | 0.075 (0.114) | 0.22 | -0.023 (0.108) | 0.34 | 0.231 (0.127) | 0.15 |
| 25 - <30 | -0.049 (0.029) | ref | 0.195 (0.110) | ref | -0.113 (0.105) | ref | 0.086 (0.121) | ref |
| 30 - <35 | -0.01 (0.03) | 0.18 | 0.09 (0.11) | 0.33 | -0.06 (0.11) | 0.58 | -0.08 (0.12) | 0.15 |
| 35+ | -0.008 (0.03) | 0.21 | -0.013 (0.12) | 0.10 | -0.095 (0.12) | 0.88 | -0.161 (0.15) | 0.09 |
| **Blood Pressure** |  |  |  |  |  |  |  |  |
| Normal | -0.01 (0.03) | ref | 0.011 (0.10) | ref | -0.01 (0.09) | ref | 0.17 (0.11) | ref |
| Pre-hypertension | -0.03 (0.04) | 0.54 | 0.02 (0.14) | 0.94 | -0.07 (0.13) | 0.62 | -0.13 (0.16) | 0.03 |
| Hypertension | -0.06 (0.03) | 0.03 | 0.23 (0.10) | 0.02 | -0.14 (0.09) | 0.13 | 0.03 (0.11) | 0.17 |
| **Diabetes** |  |  |  |  |  |  |  |  |
| No | 0.01 (0.02) | 0.02 | 0.00 (0.07) | 0.21 | -0.09 (0.07) | 0.78 | -0.06 (0.08) | 0.33 |
| Yes | -0.07 (0.04) |  | 0.17 (0.14) |  | -0.05 (0.13) |  | 0.10 (0.17) |  |

Abbreviations: TBV, total brain volume; AWM, abnormal white matter; GM-CBF, gray matter cerebral blood flow;

WM-FA, white matter fractional anisotropy.
