# Supplementary material for: Vascular Factors and Multiple Measures of Early Brain Health: CARDIA Brain MRI Study
Source: PLoS One. 2015 Mar 26;10(3):e0122138. doi: 10.1371/journal.pone.0122138 (PMC4374951; doi:10.1371/journal.pone.0122138)
Supplement: S3 Table — (DOCX) [file pone.0122138.s003.docx]

S3 Table. Association of individual tests of cognition and multiple measures of brain health: CARDIA BRAIN Sub-study.

|  | **TBV** |  | **AWM** |  | **WM-FA** |  | **GM-CBF** |  |
| --- | --- | --- | --- | --- | --- | --- | --- | --- |
|  | Effect | p | Effect | p | Effect | p | Effect | P |
| DSST (correct # digits) | 8.140 (1.943) | <.001 | -0.909 (0.525) | 0.08 | 1.683 (0.546) | 0.002 | 0.904 (0.643) | 0.16 |
| Stroop (seconds + errors) | -1.486 (1.270) | 0.24 | 0.909 (0.338) | 0.007 | -0.638 (0.354) | 0.07 | -0.263 (0.427) | 0.54 |
| Memory (words recalled) | 0.243 (0.330) | 0.46 | -0.118 (0.088) | 0.18 | 0.147 (0.092) | 0.11 | 0.234 (0.113) | 0.04 |

All models are adjusted for age, sex, race and education.
